# Exploring the Mediating Roles of State and Trait Anxiety on the Relationship between Middle Adolescents’ Cyberbullying and Depression

**DOI:** 10.3390/children7110240

**Published:** 2020-11-19

**Authors:** Ana-Nicoleta Grigore, Alexandra Maftei

**Affiliations:** Faculty of Psychology and Educational Sciences, Alexandru Ioan Cuza University, Bulevardul Carol I, Nr.11, 700506 Iaşi, Romania; grigoreananicoleta@gmail.com

**Keywords:** cyberbullying, state anxiety, trait anxiety, depression, adolescents

## Abstract

Cyberbullying is a global problem with significant negative implications, especially among more vulnerable populations, such as adolescents. Previous research suggested that cyberbullying is significantly associated with depression, and anxiety seems to partially or fully mediate this relationship. We aimed to investigate the prevalence and the relationships between cyberbullying status (i.e., cyberbully, cyber victim, double role, or non-cyber), gender, and age. We also explored the mediating roles of state and trait anxiety on the relationship between adolescents’ cyberbullying victimization, cyber-aggressiveness, and depression. Our sample consisted of 501 middle adolescents aged from 12 to 15 years (*M* = 14.00; *SD* = 0.80; 51.1% males). The results suggested no significant associations between participants’ status, gender, or age. Mediation analyses suggested that the relationship between cyber-victimization, cyber-aggressiveness, and depression was mediated by state anxiety and not trait anxiety. We discuss the implications of the current findings in understanding cyberbullying’s psychological consequences and their relevant practical implications for prevention and intervention programs.

## 1. Introduction

### 1.1. Bullying and Cyberbullying

The bullying phenomenon has been studied since the early 1970s [1,2]. Various researchers worldwide have agreed that bullying is a phenomenon increasingly present and that it has become a worrying social problem in educational, organizational, or interpersonal settings [3,4,5,6,7,8]. In recent years, with technology development, a new form of bullying emerged, namely cyberbullying, also known as electronic harassment or Internet harassment. Cyberbullying, an ongoing public health issue, is generally defined as the online (i.e., through some media—e.g., text messages, calls, social networks, or e-mails) aggressive act committed by a person or a group of people to repeatedly harm a person who cannot defend himself/herself [9]. In addition to intentionality, imbalance of power, and repetition as common features with traditional bullying, cyberbullying involves modern technology [10,11]. Features such as anonymity, a possible larger audience, and the possibility of perpetuating the aggressive act at any time, in addition to the physical distance that separates the victim from the aggressor, significantly contribute to the increasing prevalence of the cyberbullying phenomenon [12,13,14,15].

All forms of bullying imply three types of participants: aggressors, victims, and witnesses. In general, aggressors are individuals who are hurt or scare, with pleasure, people they perceive as weaker or smaller than themselves [16]. They do not like to lose, do play dirty, are aggressive, and generally have a low level of empathy [17,18]. Victims are people who are intentionally harmed by the aggressor [17]. They are often shy, introverted, or sensitive, while having low self-esteem [1]. Finally, the bystanders represent those who witness the victim’s aggression. They can, most often, encourage the aggressor, remain distant and silent, or defend the victim [18]. In the present study, we were interested in both the aggressors and the victims of cyberbullying.

Cyberbullies usually exhibit school problems (i.e., substance use and aggressive behavior [13,18,19], they have low self-esteem, show hyperactivity, they have difficulty to express emotions appropriately (e.g., anger), and usually use technology as a way to vent their frustrations [20,21]. Due to their ability to keep their identity unknown and the lack of face-to-face contact with the victim, cyberbullies are mostly unlikely to experience compassion or sympathy toward the victim [22]. On the other hand, cyberbullying victims “feel less popular, take more internet-related risks, are more often a bystander and perpetrator of internet and mobile phone bullying, and are less often a perpetrator and more often a victim of traditional bullying” ([23] p.1349). Cyberbullying victims usually express social problems and depressive symptoms, along with school refusal and various physical symptoms associated with increased psychological distress [21,24]. These adverse effects can have long-term consequences on both the victim and their family and peers [19].

### 1.2. Anxiety, Depression, and Cyberbullying

Previous research suggested that cyberbullying perpetration is significantly associated with depression, and anxiety seems to partially or fully mediate this relationship in both clinical and non-clinical populations [25,26]. High levels of depression and stress were found to increase the probability of being both a cyberbullying victim as well as a cyberbullying perpetrator [27]. Furthermore, cyber victims and cyber aggressors seem to have similar levels of depression [28]. A recent meta-analysis conducted by Marciano, Schultz, and Camerini [29] explored the longitudinal data available concerning cyberbullying perpetration and victimization in youth. Their study involved 56 longitudinal studies, and the results suggested that cyberbullying perpetration generally determines externalizing problem behaviors over time, while cyberbullying victimization represents a risk factor for depression and anxiety. Additionally, depression, anxiety, and Internet use were found to be significant cyberbullying victimization predictors over time.

Jenaro, Flores, and Frías [30] explored the medium- and long-term impacts of past experiences of cyberbullying on students, and their results suggested that students who have been cyberbullied scored significantly higher for anxiety and depression. Moreover, those students who were cyberbullied in secondary school had a significantly lower adjustment at the university level. Similar associations between cyberbullying victimization, depression, and anxiety were found in various samples of children and adolescents worldwide [31,32,33].

In line with previous findings reported by Na, Dancy, and Park [34], Wang, Xie, Wang, Lei, and Jiang [26] suggested that cyberbullying victimization predicts depression directly and indirectly, through the mediating role of social anxiety. Similarly, according to Martínez-Monteagudo, Delgado, Inglés, and Escortell [35], cyberbullying victims score highly for social avoidance and social distress. One of the most worrying effects of cyberbullying victimization is related to suicidal thinking and behavior [13,36,37,38], emphasizing the importance of cyberbullying research, prediction, and intervention strategies to promote positive mental health, especially at younger ages, such as in childhood and adolescence [39].

### 1.3. Cyberbullying, Gender, and Age

Several researchers highlighted the importance of gender in the emergence of bullying behavior [40], generally suggesting that both girls and boys engage in this type of behavior [41]. However, studies have shown that most perpetrators are males, whereas in victims, there is no link between gender and the likelihood of them becoming victims [42,43]. Von Marées and Petermann [44] suggested that males are more likely to become aggressors as their age increases, in line with similar results presented by Sentse, Kretschmer, and Salmivalli [45] or Tustin, Zulu, and Basson [46].

Barlett and Coyne [40] conducted a meta-analysis to explore whether there are significant gender differences in the occurrence of cyberbullying behaviors and the potential moderating role of age within this relationship. Their results showed that male subjects were less prone to cyberbullying behaviors than women, compared to other findings [47]. Age moderated this relationship; female participants were found to be more likely to engage in cyberbullying behaviors during early adolescence compared to male subjects, who had higher levels of cyberbullying in late adolescence, supporting other similar results in this area [48,49].

Other studies suggested no gender differences in victimization rates [50,51] and reported conflicting results. For example, Kowalski and Limber [19] suggested that girls are more likely to experience cyberbullying (as both victims and perpetrators), while other authors found no significant difference between girls and boys in neither cyberbullying perpetration nor victimization [13,14,52]. Cyberbullying behaviors (i.e., aggressors) and victimization are subject to developmental variations. Cyber aggressors’ behaviors seem to increase in adolescent ages 12 to 16 [13,53], while cyber victimization seems to decline throughout [54]. However, according to Simões and Matos [55], school satisfaction is more important for offending and teenagers with double roles (both victims and aggressors), as the subjective health complaints seem to be risk factors for all three profiles (i.e., victims, aggressors, and double roles).

### 1.4. The Present Study 

According to a recent Eu Kids Online report [56], 84% of Romanian children access the Internet through smartphones, with an average of 178 min spent online daily. Half of the youth (i.e., in the Eu Kids Online sample) reported that negative experiences increased from 21% in 2010 to 33% in 2018. In most countries, 14- to 16-year-old adolescents spend almost twice as much time online than 9- to 10-year-olds. Children and adolescents aged 9 to 16 years ranked as the highest group to access the Internet and engage in daily online activities. Romanian boys reported being victims more than girls, and out of those children who witnessed other teens being cyberbullied, half of them tried to help the victim, 45% did not react, and 7% encouraged the aggressor. Furthermore, one-third of the children reported negative comments received for personal content shared online (most of it without consent), a frequent practice in Romania [57].

Given the significant negative consequences that cyberbullying has on one’s mental health, in addition to the worrying data related to cyberbullying all over the world, Romania included, in the current research, our aims were (1) to investigate the prevalence and the relationships between cyberbullying status (i.e., cyberbully, cyber victim, double role, or non-cyber), gender, and age; and (2) to explore the mediating roles of state anxiety (i.e., the psychological and physiological reactions toward adverse situations in a specific moment; [58]) and trait anxiety (i.e., a trait of personality (therefore, stable over time) describing individual differences related to one’s tendency to present state anxiety; [59]) on the relationship between middle adolescents’ cyberbullying victimization, cyber-aggressiveness, and depression. Furthermore, we also aimed to examine whether the existing shreds of evidence suggested by fellow researchers from other countries are generalizable across other cultural samples, such as in Romania.

Based on the earlier studies discussed, we assumed the following: (H1) a high prevalence rate among cyber victims in both boys and girls [13,14,52]; (H2) higher rates of cyber-aggression among older, male boys [13,53,54]; (H3) lower rates of cyber-victimization among older participants [13,53,54]; (H4) significant associations between cyber status (i.e., cyberbully, cyber victim, double role, or non-cyber), and depression, anxiety state, and anxiety trait. Concerning our fifth assumption (H5), we expected state anxiety and trait anxiety to mediate the relationships between cyber-victimization and cyber-aggression, in line with previous studies that confirmed partial or total mediation effects of anxiety [25,26,34]. However, we were also interested in exploring which of the two anxiety dimensions has a more substantial effect on the relationship between cyberbullying and depression.

## 2. Materials and Methods

### 2.1. Participants

Our final cross-sectional sample consisted of 501 middle adolescents aged 12 to 15 years (*M* = 14.00; *SD* = 0.80; 51.1% males). They were all students in a north-eastern public school in Romania, with similar socio-economic backgrounds. The inclusion criteria were related to having and using a personal smartphone (regardless of the frequency of use). Seven participants from our initial sample were excluded due to incomplete demographic information (i.e., gender and age) or lack of responses to one of the questionnaires. Participation was voluntary and all the middle adolescents in our sample received a colorful sticker at the end of the research procedure as a reward for their participation.

### 2.2. Research Procedure

This study’s protocol was designed in concordance with the ethical requirements specific to the faculty where the authors are affiliated. All participants voluntarily participated in the study and gave written informed consent, following the 2013 Declaration of Helsinki and the national laws from Romania regarding ethical conduct in scientific research, technological development, and innovation. Before administering the questionnaire, the informed consent of the legal representatives of the adolescents (i.e., parents) was obtained. The research was conducted in late 2019, in participants’ usual classrooms, following the school principal’s approval. The questionnaires were distributed by the school psychologist and one research assistant, blind to the study’s aims.

### 2.3. Instruments

We used an adapted version of The Beck Depression Inventory-II (BDI II; Beck and Steer, [60], adapted and validated on the Romanian population by David and Dobrean, [61]) to measure depression. The scale consists of 21 items that measure depression on a scale from 0 (symptom absence) to 3 (severity of the symptom). We only used the first 20 items, given the age of our participants and the nature of the last dimension (Loss of Interest in Sex). Participants were asked to read each group of statements carefully and pick out the one statement in each group that best describes how they have been feeling during the past two weeks, including the present day. Example items included “I feel my future is hopeless and will only get worse” (a 3-score on the Pessimism dimension); “I dislike myself” (a 3-score on the Self-Dislike dimension); or “I have much greater difficulty in making decisions than I used to” (a 2-score on the Indecisiveness dimension). Cronbach’s alpha coefficient for the present study indicated high reliability of 0.915. 

We further used the State-Trait Anxiety Inventory (STAI, Spielberger [62]) to measure anxiety. A self-report questionnaire was designed to assess levels of state anxiety and trait anxiety. Participants answered each of the 40 items on a scale from 1 (almost never) to 4 (almost always). Example items for the trait anxiety subscale include “I worry too much over something that really doesn’t matter” and “I am content; I am a steady person.” For the state anxiety subscale, example items include “I am tense”; “I am worried”, and “I feel calm; I feel secure.” Higher scores indicate higher levels of anxiety. Cronbach’s alpha coefficient for the present study indicated high reliability for both the state anxiety subscale (0.920) and the trait anxiety subscale (0.893). 

Finally, we used the short 9-item form of the European Cyberbullying Intervention Project Questionnaire (ECIPQ, Brighi et al. [63]), to assess the experience of cyber victimization and cyber aggression. Participants answered a total of 18 items (nine items for each dimension, i.e., aggressor or victim) on a Likert-type scale from 1 (never) to 5 (almost daily). The experience as a victim of cyberbullying dimension explored direct behaviors (i.e., direct aggression—”Someone told me something nasty or threatened me via the Internet or texting”; posting or editing of embarrassing personal pictures or videos—“Someone posted online embarrassing pictures or videos or modified the one I posted”; identity theft—“Someone illegally logged into my e-mail or social network account and stole personal information”), or indirect aggressions, such as releasing personal information (e.g., “Someone released personal information on me online”) or spreading rumors (e.g., “Someone spread rumors about me online”). The same items were used in an active form to assess cyberbullying behaviors perpetrated by subjects. A high score on each of the two dimensions indicated a higher experience as a cyberbully or a cyber-victim. Cronbach’s alpha coefficient for the present study indicated satisfying reliability coefficients for both the cyber-victim subscale (0.856) and the cyber-aggressor subscale (0.755).

A demographic scale assessed participants’ gender and age. All instruments were first pre-tested in a similar sample of middle adolescents (*N* = 35, *M* age = 14.01), and no difficulties were reported in terms of understanding the concepts and the words used within the questionnaires.

## 3. Results

### 3.1. Cyber Status, Gender, Age 

We used the SPSS 20.0 program to analyze our data. Based on the ECIPQ subscales’ scores and the median test, participants were grouped in four cyber statuses: cyberbully, cyber victim, double role (i.e., both cyber bullies and cyber victims), or none of the above (neither a cyber victim nor a cyber aggressor). To be classified as a cyberbully, participants need to have higher scores on the cyber-aggressor subscale (>*Mdn* = 16) and low scores on the cyber victim scale (<*Mdn* = 18). Similarly, we classified participants who scored higher >*Mdn* = 18 on the cyber victim subscale and lower than 16 on the cyber aggressor dimension as cyber victims. The adolescents with a double role scored highly on both dimensions. Higher scores on both subscales implied a double role (both as a victim and a cyber aggressor). The lowest possible score on the ECIPQ subscales was 9. Therefore, we considered participants who scored nine or lower than the median on any of the two subscales as non-cybers (neither a cyber victim nor a cyber aggressor) (see Table 1). Chi-square tests indicated no significant associations between participants’ status (i.e., cyberbully, cyber victim, double role, or non-cyber) and gender (*X*^2^(3, 501) = 1.83, *p* = 0.607), nor with participants’ age (*X*^2^(3, 501) = 5.53, *p* = 0.136).

### 3.2. Cyber Status, Anxiety, and Depression

Further analyses suggested a significant association between participants’ status and state anxiety (*X*^2^(3, 501) = 173.635, *p <* 0.001). Participants with higher levels of state anxiety were more likely to be victims (*N* = 80). In contrast, participants with lower scores on the state anxiety subscale were more likely to have a double role (*N* = 96), to be aggressors (*N* = 69), or to be non-cybers (*N* = 157). We also found a significant association between participants’ status and depression (*X*^2^(3, 501) = 66.12, *p <* 0.001). Participants with higher levels of depression were more likely to be victims (*N* = 77), while participants with lower scores on the depression scale were more likely to have a double role (*N* = 102) or to be non-cybers (*N* = 91). 

Finally, a significant association was found between participants’ status and trait anxiety (*X*^2^(3, 501) = 88.89, *p <* 0.001). Participants with higher levels of trait anxiety were more likely to be victims (*N* = 72) or to have a double role (*N* = 102). In contrast, participants with lower scores on the trait anxiety subscale were more likely to be aggressors (*N* = 56) or non-cybers (*N* = 124).

### 3.3. The Mediating Role of Anxiety 

To deepen our exploration, we further performed mediation analyses using the Hayes [63,64] SPSS macro program PROCESS. The theoretical hypothesis model was tested by estimating the 95% confidence interval (CI) for mediation effects with 5000 resampled samples. In the current study, we selected Model 4 to analyze the mediating effect of state anxiety and trait anxiety on the relationship between cyber aggressiveness and cyber victimization. We used the total scores of the cyberbullying subscales (i.e., total scores for cyber victimization and cyber aggression) within the ECIPQ. 

Descriptive analyses. The means, standard deviations, and correlation matrix of each variable are shown in Table 2. We found significant positive associations between cyber victims and trait anxiety, state anxiety, and depression. Similar significant positive associations were found between cyber aggressors, state anxiety, and depression. A negative, significant association was found between cyber aggressors and depression.

**Mediation effect analysis**. Model 4 is a simple mediating model in the SPSS macro PROCESS compiled by Hayes [63]. We used it to test the mediating effect of trait and state anxiety on the relationship between cyberbullying (i.e., cyber-victimization and cyber-perpetration) and depression. 

#### 3.3.1. The Mediating Role of State Anxiety and Trait Anxiety on the Relationship between Cyber-Victimization and Depression

The results suggested a positive, significant effect of cyber-victimization on state anxiety (*b* = 1.47, *t* = 28.31, *p* < 0.001) and on trait anxiety (*b* = 1.17, *t* = 17.89, *p* < 0.001). State anxiety significantly predicted depression (*b* = 0.55, *t* = 10.18, *p* < 0.001), but trait anxiety did not have a significant predictive effect on depression (*b* = 0.06, *t* = 1.57, *p* = 0.11). The indirect effect of cyber-victimization and depression was mediated by state anxiety (indirect effect = 0.82, SE = 0.11, 95% CI = [0.61 − 1.04]) but not trait anxiety (indirect effect = 0.007, SE = 0.06, 95% CI = [−0.004 − 0.019]). 

#### 3.3.2. The Mediating Role of State Anxiety and Trait Anxiety on the Relationship between Cyber-Aggressiveness and Depression

The results suggested a positive, significant effect of cyber-aggressiveness on state anxiety (*b* = 0.63, *t* = 4.74, *p* < 0.001) but not on trait anxiety (*b* = 0.15, t = 1.17, p = 0.24). State anxiety significantly predicted depression (*b* = 0.62, *t* = 15.00, *p* < 0.001), but trait anxiety did not have a significant predictive effect on depression (*b* = 0.03, *t* = 0.80, *p* = 0.42). The indirect effect of cyber- aggressiveness and depression was mediated by state anxiety (indirect effect = 0.39, SE = 0.07, 95% CI = [0.259 − 0.054]) but not trait anxiety (indirect effect = 0.005, SE = 0.01, 95% CI = [−0.016 − 0.040]). 

## 4. Discussion

Our primary findings suggested no significant associations between participants’ status, gender, or age. Therefore, H1, H2, and H3 were not confirmed. We assumed that our data would suggest significant associations between cyber status (i.e., cyberbully, cyber victim, double role, or non-cyber), depression, anxiety state, and anxiety trait (H4). The results confirmed this assumption, suggesting that (a) participants with higher levels of state anxiety were more likely to be victims, while participants with lower scores on the state anxiety subscale were more likely to have a double role, to be aggressors, or to be non-cybers; (b) participants with higher levels of depression were more likely to be victims, and adolescents with lower scores for depression were more likely to have a double role or to be non-cybers; (c) middle adolescents with higher levels of trait anxiety were more likely to be victims or to have a double role, while participants with lower scores for trait anxiety were more likely to be cyber aggressors or to be non-cybers. Mediation analyses suggested that the relationship between cyber-victimization, cyber-aggressiveness, and depression was mediated by state anxiety and not trait anxiety. 

The lack of significant associations between gender, age, and cyber status seem to confirm previous results concerning this area of research (i.e., [19,50,51]), which might be explained by the similar percentages in both girls and boys in this specific age group (12 to 15 years) concerning cyberbullying exposure, victimization, and perpetration. However, further studies are needed to better clarify the conflicting results concerning cyberbullying, age, and gender. Results concerning the associations between cyber status and anxiety trait, anxiety state, and depression are probably our most important findings, with relevant practical implications. First of all, we found that middle adolescents with higher levels of depression and state and trait anxiety were more likely to be cyber-victims, confirming previous findings in this area (e.g., [29]). This is an essential insight for future prevention and intervention programs that would benefit from focusing on cultivating adolescents’ well-being and emotional balance. Second, mediation analyses suggested the significant mediating role of state anxiety, which adds to the importance of enhancing adolescents’ well-being. We already know that the most frequently used intervention in the area of cyberbullying generally includes education on coping skills, empathy training, social skills, and communication training [65,66]. However, in line with similar previous findings [39], our results point out the usefulness of intervention programs aimed at increasing adolescents’ well-being and to reduce psychological distress and, implicitly, cyberbullying, such as cognitive behavioral programs [66,67,68], along with social support [69] and positive family and peer relationships [70,71].

Children and adolescents generally spend time online on social media platforms. As we already know, there might be a direct relationship between anxiety, depressive symptoms, and social media use [72,73]. Hunt, Marx, Lipson, and Young [74] suggested that limiting social media use might decrease loneliness and depression. Furthermore, spending less time online reduces the risks of becoming a cyber victim and continuing to be a cyberbully. Therefore, intervention strategies in both school and family settings should also aim to reduce the time spent online by children and teenagers and replace it with valuable, family and peer offline time.

A series of limitations also need to be addressed. First, all measures were self-reported, which might have generated biased responses. Second, the school setting might have inhibited participants and, therefore, it might have also increased the likelihood of desirable answers. Third, we did not account for other variables that were found to be significant for cyberbullying among middle adolescents, such as difficulties in emotion regulation and loneliness, sexual orientation [75], empathy and moral disengagement [76], and several other individual, family, and neighborhood characteristics [77].

Despite these limitations, we consider the current findings relevant for therapeutical, school, and family-based intervention strategies to prevent, reduce, and eradicate the risk factors and adverse effects of cyberbullying.

## Figures and Tables

**Table 1:** Participants’ status (i.e., cyberbully, cyber victim, double role, or non-cyber) depending on gender and age (*N* = 501).

	Gender	*N*
Aggressor	Male	39
(15%) *	Female	36
Victim	Male	43
(18.6%) *	Female	50
Double role	Male	76
(30.5%) *	Female	77
Non-cybers	Male	98
(35.9%) *	Female	82

* Note: Percentages are calculated from the total sample of participants (*N* = 501).

**Table 2:** Means, standard deviations, and correlations between the main variables (*N* = 501).

Variables	*M*	*SD*	1	2	3	4	5
1. Cyber victim	18.71	6.61	1	0.324 **	0.785 **	0.625 **	0.516 **
2. Cyber aggressor	16.29	4.07	0.324 **	1	0.208 **	0.052	−0.095 *
3. State anxiety	39.99	12.42	0.785 **	0.208 **	1	0.673 **	0.650 **
4. Trait anxiety	44.56	12.44	0.625 **	0.052	0.673 **	1	0.478 **
5. Depression	13.97	11.48	0.516 **	−0.095 *	0.650 **	0.478 **	1

* *p* < 0.005; ** *p* < 0.001.
